# Hypertension Prevalence and Associated Risk Factors in a South African Population

**DOI:** 10.3390/ijerph23040514

**Published:** 2026-04-16

**Authors:** Hannah Fokkens, Jyoti R. Sharma, Ria Laubscher, Teke Apalata, Samuel Y. Alomatu, Hans Strijdom, Rabia Johnson

**Affiliations:** 1 Biomedical Research and Innovation Platform, The South African Medical Research Council (SAMRC), Cape Town 7501, South Africa; jyoti.sharma@mrc.ac.za (J.R.S.); rabia.johnson@mrc.ac.za (R.J.); 2 Centre for Cardio-Metabolic Research in Africa, Division of Medical Physiology, Faculty of Medicine and Health Sciences, Stellenbosch University, Cape Town 7501, South Africa; jgstr@sun.ac.za; 3 Biostatistics Research Unit, The South African Medical Research Council (SAMRC), Cape Town 7501, South Africa; rialaubscher62@gmail.com; 4 Division of Medical Microbiology, Department of Laboratory-Medicine and Pathology, Faculty of Health Sciences, Walter Sisulu University and National Health Laboratory Services, Mthatha 5099, South Africa; tapalata@wsu.ac.za (T.A.); samalomatu@yahoo.com (S.Y.A.)

**Keywords:** hypertension, cardiovascular diseases, risk factors, South Africa, rural health, public health intervention

## Abstract

**Highlights:**

**Public health relevance—How does this work relate to a public health issue?**

- This study addresses hypertension, a leading risk factor for cardiovascular disease, by documenting its high prevalence and key determinants in a South African community.
- It links individual lifestyle and biological risk factors to broader socioeconomic and rural health disparities.

**Public health significance—Why is this work of significance to public health?**

- The high prevalence of hypertension highlights an urgent public health challenge requiring strengthened prevention and control strategies.
- Identification of modifiable risk factors supports targeted interventions to reduce cardiovascular disease burden and health inequities.

**Public health implications—What are the key implications or messages for practitioners, policy makers and/or researchers in public health?**

- Findings support improved hypertension screening, integrated chronic disease management, and community-based lifestyle interventions, particularly in rural areas.
- These results highlight priorities for policy and future research aimed at improving healthcare access and addressing social determinants of health.

**Abstract:**

Worldwide, hypertension is a major risk factor for stroke and cardiovascular diseases, creating serious public health issues. This study aimed to evaluate the risk factors and the prevalence of hypertension in a community in South Africa. Between 2019 and 2023, an observational study with 1029 participants was carried out. The South African Hypertension Society hypertension guidelines were used to determine the prevalence of hypertension. Risk factors, such as anthropometric factors, socioeconomic factors, and lifestyle choices, were evaluated using univariate and multivariate logistic regression analysis. Within the total study population, the mean age was 48 years, and 81.1 percent of the participants were female. The mean blood pressure was 128/82.5 mmHg and 48.7% of the participants were obese. The prevalence of hypertension was 53.6%. Significant risk factors for hypertension included ageing, diabetes, having a higher body mass index, not having formal education, being unemployed, leading a sedentary lifestyle, and living in a rural area. The study highlights the increased prevalence of hypertension in this South African population. The findings were consistent with the current literature with regard to hypertension risk factors, such as age, body mass index, education, and physical activity. Current data highlights the need for focused health education and awareness initiatives that encourage healthy living. Improving healthcare access and addressing the socioeconomic factors should be the main goals of policy initiatives to lessen the impact of hypertension in underprivileged rural communities.

## 1. Introduction

According to the World Health Organization (WHO), cardiovascular diseases (CVDs) are the number one cause of all global deaths [1]. Of these deaths, 85% are due to heart attack and stroke, with hypertension (HTN) being the number one risk factor [1]. HTN, defined by a blood pressure reading of ≥140/90 mmHg, is reported to affect 1.28 billion adults, aged 30–79 years [2,3]. Furthermore, HTN is said to be more prevalent in low-to-middle-income countries (LMICs) as compared to high-income countries, with the African region having among the highest prevalence [3]. Several studies have reported that this epidemiological transition in HTN prevalence from high-income countries (HICs) to LMICs was driven by various factors including rapid urbanisation, lifestyle changes, and health disparities with varying levels of healthcare access and inequalities [4,5].

Africa has been observed to have the highest HTN prevalence of the WHO regions, accounting for 27% of cases [1]. In a systematic review by Olowoyo and colleagues (2025), the authors reported an HTN prevalence of 28.5% in Africa during 2002–2023 [6]. Of concern was the review by Gafane-Matemane and colleagues (2024), who reported on a HTN prevalence of 35% in sub-Saharan Africa during 2017–2023 [3]. South Africa, an upper-middle-income country, is one of the countries that has observed a substantial shift in lifestyle and behaviour changes in the last two decades. This shift, coupled with its quadruple burden of diseases, has contributed to the higher prevalence of HTN compared to other sub-Saharan African countries [7]. In a cross-sectional study done by Woodiwiss and colleagues (2023) during 2017–2021, the authors reported that South Africa had an HTN prevalence of 29.7%, with more men than women being hypertensive [8]. Of greater concern is that individuals living in rural parts of South Africa are experiencing a sociocultural transition towards a westernised lifestyle, which has been identified as the primary factor driving the increase in HTN prevalence in these areas. Of note, the increase has elevated HTN prevalence in rural parts of sub-Saharan Africa to levels comparable to those observed in urban populations [3,9,10]. Additionally, it has been reported that the odds of being hypertensive are further exacerbated by the reach of healthcare facilities and the rising prevalence of diabetes and obesity in both rural and urban areas [11,12,13]. Together, these factors contribute to the growing burden of HTN, complicating efforts to better manage, detect and reduce its prevalence.

To explore this further, the current study seeks to evaluate the prevalence and determinants of HTN within rural areas in South Africa and further aims to elucidate the impact of the SAHS guidelines on HTN prevalence.

## 2. Materials and Methods

### 2.1. Study Population

**Figure 1:**
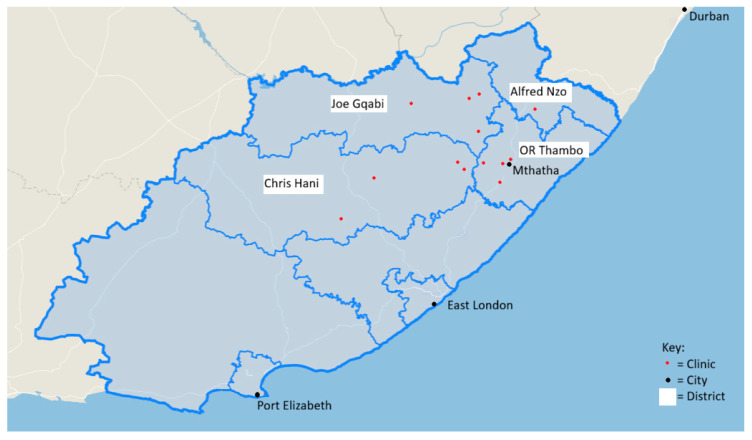
Location of 14 study clinics from 4 districts in Eastern Cape, South Africa. Adapted from Census 2011 (accessed on 13 June 2023, https://census2011.adrianfrith.com/place/294040).

In total, 1029 isiXhosa-speaking participants aged 18 years and older were recruited from 4 health districts (OR Tambo, Alfred Nzo, Chris Hani, and Joe Gqabi) and 14 community healthcare facilities in the Eastern Cape Province of South Africa over a period of 4 years (23 May 2019–20 November 2023) (Figure 1). A facility-based convenience sampling approach was used. Healthcare facilities were selected to ensure representation across districts and rural–urban settings. Within each facility, all eligible individuals attending the clinic during data collection periods were invited to participate. Participants were enrolled consecutively based on willingness to provide informed consent. Sampling was therefore not random and was not conducted in proportion to the facility’s patient volume. As a result, the sample may not be fully representative of the underlying population.

The OR Tambo district has the highest population density of 112 people per km^2^, followed by Alfred Nzo, Chris Hani and lastly Joe Gqabi with a density of 13 people per km^2^. Of the community healthcare facilities involved in this study, 7 were situated in rural areas as defined by the Rural Development Framework. The rural areas are characterised as sparsely populated areas in which people depend on natural resources or farming, including villages and small towns dispersed throughout these areas. Urban and rural classification was based on the location of the healthcare facilities where participants were recruited. Facilities were categorised according to the South African Rural Development Framework.

### 2.2. Eligibility Criteria

The study included participants aged 18 years and older, excluding pregnant females and individuals who did not provide consent or presented with a fever or cough at the time of recruitment.

### 2.3. Clearance

The study was approved by the ethics committees of the South African Medical Research Council (EC028-8/2020); Stellenbosch University Human Research (S22/05/095); Eastern Cape Department of Health (EC_2017RP56_979); and Walter Sisulu University (073/15). The study complied with the ethics guidelines as outlined in the Declaration of Helsinki [14]. Important information on the participants’ rights, the objective of the research, and the research procedure was provided to participants in both English and isiXhosa before they signed an informed consent form.

The appropriate sample size was calculated using the following formula:

$$n\;=\;\frac{p\left(1-p\right)z^{2}}{d^{2}}=\;\frac{0.30\left(1-0.30\right)1.96^{2}}{0.05^{2}}\;=322$$

where z is the confidence level, *p* is the expected proportion of patients with HTN and d is the margin of error, with *p* set at 0.30; the desired precision is 5%. The calculation was done at a 95% confidence level. A total of 1029 participants were included to compensate for incomplete records.

### 2.4. Data Collection

Data and consent forms were entered into the Research Electronic Data Capture system (REDCap™) using a modified version of the WHO STEPwise questionnaire. The collected data encompassed anthropometric measurements (waist circumference (WC), hip circumference (HC), height, and weight) as well as sociodemographic variables (age, income, education, employment, dietary habits, smoking, alcohol intake, exercise habits, family history of diabetes, HTN, and dyslipidaemia). Human immunodeficiency virus (HIV) status was documented exclusively for participants who were willing to disclose their status. HIV status was documented on a voluntary basis due to its sensitive nature. As a result, this variable contained missing data and was not included in the regression analyses.

### 2.5. Collection of Sociodemographic and Behavioural Data

Participants self-reported various factors including education, income, employment, dietary habits, smoking, alcohol consumption, family history of HTN or diabetes, and exercise habits. Income was categorised into two groups, <R2500 and ≥R2501 per month. Education was recorded based on categorial variables: none, primary, secondary, and tertiary. Employment status was categorised as unemployed and employed. Smoking status was self-reported and participants were categorised as current smokers or non-smokers; participants who reported exposure to second-hand smoke were included in the smoker category. Alcohol consumption was self-reported and recorded as a binary variable (current use vs. no use). More than >70% of participants reported no smoking and no alcohol intake, which is contradictory to previous reports from the same region, suggesting potential underreporting and misclassification bias [15]. This information is sensitive, and with the potential influence of stigma, it is likely that self-reported information was inaccurate; therefore, these variables were not included in the further analysis due to concerns regarding data reliability. Exercise habits were categorised based on WHO recommendations of 150 min of physical activity per week.

All the anthropometric measurements, including the measurement of height and weight and the calculation of the body mass index, were performed using standardised equipment across study sites according to standard operating procedures (SOPs). Beam balance scales and mounted stadiometers were routinely checked for accuracy prior to use. Fieldworkers underwent training before data collection to ensure consistency and adherence to standard measurement protocols.

Measurements of height and body weight were obtained to calculate the conicity index (CI), waist-hip ratio (WHR), and body mass index (BMI). Participants were weighed to the nearest 0.1 kg while wearing light clothing and being barefoot using a traditional beam balance. A mounted stadiometer was used to measure height to the closest 0.1 cm. The BMI was calculated via the subsequent formula: weight/(height)^2^, following the WHO standard BMI classifications (<18.5 kg/m^2^ underweight, 18.5–24.9 kg/m^2^ normal weight, 25.0–29.9 kg/m^2^ overweight, and >30 kg/m^2^ obese) [16]. The WC measurement was taken at approximately halfway between the lower edge of the last palpable rib and the top of the iliac crest [16]. The measurement of the HC was taken around the buttocks’ widest point. The following formula was used to obtain the waist-hip ratio: waist circumference (units)/hip circumference (units). The conicity index was calculated as: waist circumference (m)/[0.109 × √(weight (kg)/height (m))]. Obesity profiles were derived from BMI, WHR and CI. Diabetes status was classified as normal (<5.6 mmol/L), prediabetic (5.6–6.8 mmol/L) or diabetic (≥6.9 mmol/L).

### 2.6. Measurement and Definition of Blood Pressure

All blood pressure devices used in the study were validated automated monitors and were used consistently across study sites according to standard operating procedures (SOPs). Devices were periodically checked for accuracy in accordance with the manufacturer’s guidelines. Before the commencement of the study, samplers were trained in accurate blood pressure measurement techniques using validated blood pressure monitoring machines. Participants were advised to sit at rest for around 5 min before blood pressure was measured. Blood pressure was monitored 3 times at 5 min intervals using a validated automated blood pressure monitor (mild touch ST-401 Nobel Supplies Group LLC. (Slight Touch), New York, NY, USA). The analysis was done on the mean of the 2nd and 3rd blood pressure reading. The classification of HTN status was based on the SAHS guidelines (Table 1) [17]. The classification of blood pressure was based on a systolic blood pressure (SBP) ≥ 140 mmHg and/or diastolic blood pressure (DBP) ≥ 90 mmHg, or medical records were assessed for current use of prescribed antihypertensive medication at the time of data collection. In accordance with SAHS guidelines, these individuals were classified as hypertensive regardless of their measured blood pressure. In accordance with these guidelines, to assess HTN prevalence, the participants were categorised and compared as normotensive or hypertensive based on BP measurements or being on antihypertensive medication.

### 2.7. Statistical Analysis

Clinical and anthropometric parameters were summarised as the mean ± standard deviation (SD) if normally distributed and as median (Q1–Q3) if skewed. Shapiro–Francia W’s test was used to assess normality. In the case of normally distributed parameters, group differences between hypertensive and normotensive groups were evaluated using Student’s t test for normally distributed variables and median regression for skewed variables. Univariate logistic regression analyses were initially performed to assess associations between individual variables and the outcome. Variables significantly associated with hypertension were subsequently included in a multivariate logistic regression model to estimate adjusted effects and calculate risk scores. These were represented as odds ratios and 95% confidence intervals, and *p*-values of ≤0.05 were considered statistically significant. Statistical analysis was performed using STATA16 (Software for Statistics and Data Science, package SE).

## 3. Results

In this observational study, a total of 1029 participants were recruited over a period of 3 years (2020–2023). Table 2 presents the clinical, anthropometric and sociodemographic characteristics of the study participants. The mean age of the recruited participants was 48.8 years and 81.1% of the participants were female. As the distribution of participants was not balanced regarding gender (81.1% females and 19.9% males), the subsequent stratified analysis was performed according to HTN status.

**Figure 2:**
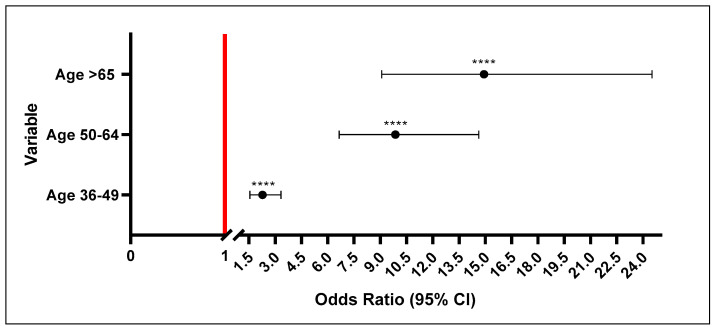
Risk scores of age groups with hypertension. The 18–35 age group was used as the reference group. The participants in the 36–49, 50–64, and >65 age groups had almost double, 5.4-fold, and 7.8-fold higher risks, respectively, of developing HTN. **** = *p* <0.001.

The prevalence of HTN according to the SAHS guidelines among the total sample (1029 participants) was 53.6% (Table 2). The mean BP was 128/82.5 mmHg and 48.7% of participants were obese. The mean blood glucose reading was 5.4 mmol/L and 58% of the study population was classified as not being diabetic, with 19.4% being classified as diabetic. There was a significant difference in HTN prevalence across various age groups (*p* < 0.001) (Figure 2). The prevalence of HTN was the highest in (81.1%) in the ≥65 years age group followed by the 50–64 age group with 74.1%, As expected, the lowest prevalence of HTN was recorded among young participants aged 18–35 years. Some variables did not show any significant association with HTN including gender (*p* = 0.96), low-density lipoprotein (LDL) cholesterol (*p* = 0.077), and high-density lipoprotein (HDL) cholesterol (*p* = 0.830). However, total cholesterol (*p* = 0.022), triglycerides (*p* = 0.019) demonstrated a significant association with HTN, reflecting variability in association across lipid parameters. Other clinical and anthropometric variables, including blood glucose (*p* < 0.001), diabetic status (*p* < 0.001), BMI (*p* < 0.001), waist circumference (*p* < 0.001), hip circumference (*p* < 0.001), waist-hip ratio (*p* < 0.001), conicity index (*p* < 0.001), showed a significant association with HTN. The prevalence of HTN was significantly higher among participants who did not perform physical activity (66.9%; *p* < 0.001). Considering the sociodemographic variables, the HTN prevalence was higher among participants who received no education (71.4%) or primary education (65.9%) as compared to those who obtained secondary (45.9%) or tertiary education (51.7%) (*p* < 0.001). Furthermore, the prevalence of HTN was significantly higher among the unemployed participants (60.1%) as compared to those who were employed (41.6%; *p* < 0.001). Regarding monthly income, 38.4% of participants earned <R2500 a month and 52.8% earned ≥R2501 a month. HTN prevalence was slightly higher (58.6%) among the participants from the lower-income group as compared those from the higher-income group (42.6%, *p* < 0.001). In rural areas, more than half of the participants were hypertensive (56.7%); in contrast, in peri/urban areas, the prevalence of HTN was lower (47.6%, *p* = 0.004).

To assess the relative risk associated with each variable, odds ratios with 95% confidence intervals were calculated. Sex was not significantly associated with hypertension in the univariate analysis (*p* > 0.05). Figure 2 depicts the odds of developing HTN among various age groups, with participants aged 18–35 years serving as the reference category. The participants aged 36–49 years had more than double (*p* < 0.001, OR = 2.3; 95% CI: 1.5–3.3) the risk of developing HTN as compared to the reference group. Interestingly, those aged 50–64 years and >65 years showed 9.8-fold (*p* < 0.001, OR = 9.8; 95% CI: 6.6–14.6), 14.9-fold (*p* < 0.001, OR = 14.9; 95% CI: 9.1–24.5) higher risks of developing HTN, respectively.

**Figure 3:**
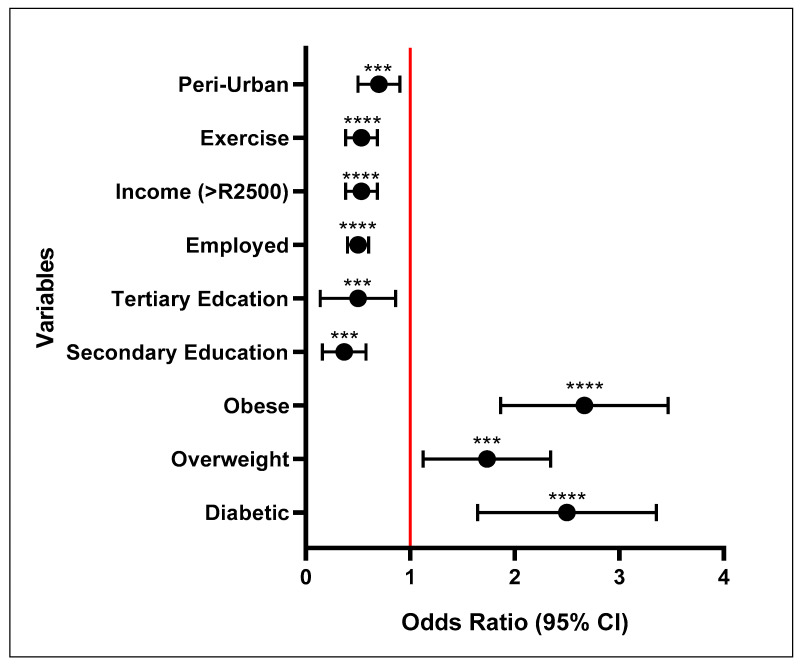
Risk scores of variables significantly increasing/decreasing the risk of developing hypertension according to the South African Hypertension Society guidelines. **** = *p* < 0.001, *** = *p* < 0.01.

Figure 3 shows that diabetic individuals had 2.4-fold (*p* < 0.001; OR = 2.4; 95% CI: 1.7–3.4) higher odds of developing HTN compared to the reference group (non-diabetic). Being overweight (*p* = 0.003, OR = 1.6; 95% CI: 1.2–2.4) and obese (*p* < 0.001, OR = 2.6; 95% CI: 1.9–3.5) increased the odds of developing HTN by 1.6- and 2.6-fold, respectively, compared with individuals of normal BMI.

Higher education levels have a protective effect on the risk of developing HTN, and participants with secondary and tertiary education had significantly lower odds of developing HTN (*p* = 0.002, OR = 0.3; 95% CI: 0.2–0.6, *p* = 0.023, OR = 0.4; 95% CI: 0.2–0.9) compared to those with no formal education. Employment was also associated with reduced odds of developing HTN (*p* < 0.001, OR = 0.5; 95% CI: 0.4–0.6) in comparison to unemployment. Physical activity decreased the risk of developing HTN (*p* < 0.001, OR = 0.51; 95% CI: 0.35–0.72), compared to those who did not exercise. Having an income of ≥R2501 decreased the risk of developing HTN (*p* < 0.001, OR = 0.5; 95% CI: 0.4–0.7), compared to those whose income was <R2500.

## 4. Discussion

In South Africa and globally, HTN serves as a major risk factor for stroke and associated cardiovascular disorders, which poses a significant public health concern [3]. It is an independent risk factor for premature mortality across all modifiable causes. The lack of knowledge regarding HTN prevalence, management, and potential causes of HTN in remote settings further exacerbates the burden of the disease [9]. As such, urgent action is required to collect health data on HTN prevalence and its risk factors for the formulation of improved community-based interventions.

This study examined the prevalence of HTN and its associated risk factors within the South African population. According to the SAHS guidelines, the prevalence of HTN is 53.6%. The total prevalence of HTN in the current study is comparatively higher (3.6% increase) than the previous study conducted between 2020 and 2021 in the same population. This could be due to the larger sample size and is consistent with globally reported increases in HTN prevalence [9]. When compared to previous studies from other provinces within South Africa, the current study reports a notably higher prevalence of HTN. For instance, earlier studies in rural and semi-urban areas of Limpopo reported a prevalence of 21% [17], 38.9% prevalence was reported in an urban population of Cape Town [18], and 41% prevalence was reported in a rural community in Limpopo [19]. In contrast, the present study found a prevalence of 53.6%. Similarly, two national surveys, the South African National Health and Nutrition Examination Survey (SANHANES) in 2012 and the South African Demographic and Health Survey (DHS) in 2016, reported prevalences of 38.4% and 48.2%, respectively, both lower than the prevalence rate observed in the current study [10]. The increase in prevalence of HTN observed in the current study compared to previous research underscores the dynamic nature of health conditions and the need for continuous monitoring and adaptation of healthcare strategies.

The current study identified a range of risk factors associated with HTN, including clinical and anthropometric factors (age, blood glucose, diabetic status, BMI group, WC, HC, WHR, CI, total cholesterol, triglycerides, SBP, DBP), as well as sociodemographic factors (education, employment, monthly income, and living conditions). These findings are supported by numerous studies across diverse populations globally [20,21,22,23] and are further supported by other studies conducted in South Africa [3,18,19], reinforcing the understanding that HTN is a multifactorial disease shaped by complex interactions between biological environmental and sociodemographic factors.

No significant gender-related risks were observed in the current study; however, these findings should be interpreted with caution due to the overrepresentation of female participants (80%). Gender disparities have been reported previously, with females presenting a significantly higher risk of developing HTN in certain populations [2,24,25]. The authors noted that other studies conducted in South Africa also reported higher participation from females when conducting population-based studies [18,19,26]. As recruitment was facility-based and relied on voluntary participation, males may be underrepresented. Although sex was not significantly associated with hypertension outcomes, the imbalance may still limit the generalisability of findings to the broader population. Future studies should consider population-based sampling or the application of post-stratification weighting to improve representativeness.

Although the current study recruited few individuals aged 65 years and older, the findings are consistent with the literature reporting that the advanced age is associated with a higher risk of developing HTN [26,27]. The limited representation of this age group could be due to reduced mobility, socioeconomic constraints, and the inability to access healthcare facilities. The findings are supported by studies that showed that with advanced age, there is an increased risk of developing HTN, potentially due to a rise in vascular resistance [27,28,29].

This study observed that physical activity was significantly associated with a decreased risk of HTN. Previously, randomised controlled trials showed that increased physical activity and reduced salt intake reduced BP [30,31,32]. Large-scale studies and randomised controlled studies also showed that weight reduction, increased physical activity, and decreased salt and alcohol intake may be effective in preventing and managing HTN [30,31,32,33]. However, a limitation of the current study was the use of the WHO STEPwise amended questionnaire, which relies on self-reported data collection regarding dietary and lifestyle habits. Due to the inconsistency of self-reported data, the salt intake, smoking and alcohol intake data were excluded from further analysis. These findings did not align with results from previous studies on the same population or with global evidence [3,34,35]. Although these factors are recognised confounders in hypertension research, they were omitted due to concerns regarding data reliability and potential underreporting likely influenced by social desirability bias. This exclusion may result in residual confounding. Similarly, HIV status was collected on a voluntary basis and contained missing data, precluding its inclusion in multivariable models. Given the potential association between the HIV infection, antiretroviral therapy, and cardiovascular risk, its omission may also contribute to residual confounding.

Risk scores of variables were assessed, and it was found that increasing age, being diabetic, being overweight and obese, having no formal education, and being unemployed increase the risk of developing HTN.

Diabetes increased the risk of having HTN by 1.9-fold in the studied population. This is consistent with previous studies that reported an overlap in the aetiology of diabetes and HTN [36,37]. Furthermore, the high prevalence of non-communicable diseases (NCDs) in South Africa’s urban and rural areas is a critical public health concern [38,39].

The most concerning finding of our study was the high prevalence of obesity and the progressively increased risk of developing HTN with rising BMI. NCDs such as diabetes, HTN, and obesity are increasing rapidly in sub-Saharan Africa [40]. This trend is greatly due to the globalisation and the increasing adoption of Westernised dietary patterns, evidenced by the rapid expansion of fast-food chains in South Africa and semi-urban and rural areas of sub-Saharan Africa [41]. A widespread shift towards Westernised diet in sub-Saharan Africa has significantly contributed to the increasing prevalence of obesity, which significantly amplifies the risk of HTN in these populations [42].

Moreover, many studies have demonstrated that a low socioeconomic status (SES) is a strong determinant of HTN risk, and our study results therefore reflect a similar trend, with factors such as no education, being unemployed, and living in a rural area significantly increasing the risk of HTN [43,44,45]. However, research has indicated that a sedentary lifestyle and excessive intake of processed foods may put higher-income groups at risk for HTN [46]. However, some studies have demonstrated that low socioeconomic status (SES) strongly influences HTN risk [36,37,38]. People living in low-SES conditions may not have access to medical services or be unable to afford costly, nutritious foods [47]. Additionally, low education levels have been shown to greatly impact disease risk as well as treatment and control of HTN [44,48,49]. The current study highlights the low levels of education and lack of access to healthcare facilities in rural areas in the Eastern Cape Province of South Africa. Although this is of great concern for the region, the factors discussed above are considered modifiable; thus, policymakers should invest timeously in education and health awareness campaigns, focusing on healthy eating and the importance of physical activity. A limitation of this study included the collection of sociodemographic data utilising self-reported data collected via the WHO STEPWise amended questionnaire. Future research should focus on developing and evaluating effective HTN prevention and management strategies for rural South African populations. This study employed a facility-based convenience sampling strategy, which may introduce selection bias, as participation was limited to individuals who attended healthcare facilities and consented to take part. Consequently, the findings may not be fully generalisable to the broader population within the Eastern Cape Province. However, the strength of this study is its inclusion of healthcare clinics in both rural and urban/peri-urban areas, which resulted in the study population having diverse socioeconomic statuses. Future studies using probability-based sampling approaches should strengthen population-level inference.

## 5. Conclusions

This study highlights an increase in the prevalence of hypertension in the Eastern Cape Province of South Africa between 2020 and 2023. This increase may be attributed to a combination of demographic, behavioural, and healthcare-related factors. Established risk factors such as increasing age, diabetes and obesity were associated with a higher risk of HTN in the study population. These findings underscore the need for developing and implementing targeted interventions to reduce the prevalence of HTN in South African populations. These interventions should prioritise the promotion of healthy lifestyles and ensure access to affordable and effective healthcare services. Additional studies are also required to investigate the underlying mechanisms of HTN in this context and identify population-specific risk factors that may inform the development of new therapeutic targets.

## Figures and Tables

**Table 1:** South African Hypertension Society guidelines for the diagnosis of hypertension.

Blood Pressure Category	SBP (mmHg)		DBP (mmHg)
Normal	<120	And/or	<80
Optimal	120–129	And	<80
High Normal	130–139	Or	80–89
Hypertension			
Grade 1	140–159	Or	90–99
Grade 2	160–179	Or	100–109
Grade 3	>180	Or	>110

BP—blood pressure; DBP—diastolic blood pressure; mmHg—millimetres mercury; SBP—systolic blood pressure. Adapted from Seedat et al., 2014 [16].

**Table 2:** Clinical, anthropometric and sociodemographic determinants of hypertension.

		Normal BP	Hypertensive	Total	*p*-Value
Sex	Female	385 (46.7%)	440 (53.1%)	825 (100%)	0.960
	Male	90 (46.9%)	102 (53.1%)	192 (100%)	
Age	18–35	183 (77.5%)	53 (22.5%)	236 (100%)	<0.001
	36–49	182 (60.3%)	120 (39.7%)	302 (100%)	
	50–64	84 (25.9%)	240 (74.1%)	324 (100%)	
	>65	31 (18.8%)	134 (81.2%)	165 (100%)	
Blood glucose		5.2 (4.7–5.9)	5.5 (4.8–7.0)	5.4 (4.7–6.3)	<0.001
Diabetic status	No	304 (51.6%)	285 (48.4%)	589 (100%)	<0.001
	Prediabetic	110 (48.0%)	119 (52.0%)	229 (100%)	
	Diabetic	60 (30.5%)	137 (69.5%)	197 (100%)	
BMI group	Normal	164 (61.7%)	102 (38.3%)	266 (100%)	<0.001
	Overweight	124 (48.8%)	130 (51.2%)	254 (100%)	
	Obese	188 (38.1%)	306 (61.9%)	494 (100%)	
WC (cm)		91.0 (80.0–102.0)	102.0 (93.0–112.0)	98.0 (86.0–120.0)	<0.001
HC (cm)		108.0 (98.0–118.0)	112.0 (102.0–122.0)	110.0 (100.0–120.0)	<0.001
WHR		0.8 (0.8–0.9)	0.9 (0.8–1.0)	0.9 (0.8–0.9)	<0.001
Conicity Index		1.2 (1.2–1.3)	1.3 (1.3–1.4)	1.3 (1.2–1.4)	<0.001
Total cholesterol		4.1 (3.4–4.5)	4.3 (3.5–4.9)	4.1 (3.5–4.8)	0.022
Triglycerides		1.1 (0.8–1.6)	1.2 (0.9–1.8)	1.1 (0.8–1.6)	0.019
HDL cholesterol		1.2 (1.0–1.4)	1.2 (1.0–1.4)	1.2 (1.0–1.4)	0.830
LDL cholesterol		2.3 (1.7–2.8)	2.4 (1.9–2.9)	2.3 (1.8–2.8)	0.077
Systolic BP		119.6 (110.5–125.7)	140.3 (129.3–155.0)	128.0 (118.0–114.7)	<0.001
Diastolic BP		78.3 (72.0–82.7)	89.3 (80.7–96.3)	82.5 (75.7–90.0)	<0.001
Education	None	12 (28.6%)	30 (71.4%)	42 (100%)	<0.001
	Primary	95 (34.1%)	184 (65.9%)	279 (100%)	
	Secondary	288 (54.1%)	244 (45.9%)	532 (100%)	
	Tertiary	85 (48.3%)	91 (51.7%)	176 (100%)	
Employment	Unemployed	261 (39.9%)	393 (60.1%)	654 (100%)	<0.001
	Employed	219 (58.4%)	156 (41.6%)	375 (100%)	
Monthly Income	<R2500	285 (41.3%)	404 (58.6%)	689 (100%)	<0.001
	>R2501	195 (57.4%)	145 (42.6%)	340 (100%)	
Physical activity	No	55 (33.1%)	111 (66.9%)	166 (100%)	<0.001
	Yes	425 (49.2%)	438 (50.8%)	863 (100%)	
Living conditions	Rural	285 (43.3%)	373 (56.7%)	658 (100%)	0.004
	Urban/peri	195 (52.6%)	176 (47.4%)	371 (100%)	

Significance was determined at *p* ≤ 0.05 for a given parameter.

## Data Availability

The data generated in this study are available from the corresponding author upon request. If you get no response from the corresponding author, please contact the Principal Investigator Rabia Johnson for any correspondence.

## Abbreviations

The following abbreviations are used in this manuscript:

HTN	Hypertension
SAHS	South African Hypertension Society
BP	Blood pressure
BMI	Body mass index
WHO	World Health Organization
CVD	Cardiovascular diseases
LMICs	Low- and middle-income countries
HICs	High-income countries
REDCap	Research Electronic Data Capture
HIV	Human immunodeficiency virus
CI	Conicity index
WHR	Waist hip ratio
Kg	Kilogram
Cm	Centimetre
WC	Waist circumference
HC	Hip circumference
SBP	Systolic blood pressure
DBP	Diastolic blood pressure
SD	Standard deviation
HDL	High-density lipoprotein
LDL	Low-density lipoprotein
CI (95%)	95% Confidence interval
SANHANES	South African National Health and Nutrition Examination Survey
DHS	Demographic and Health Survey
NCDs	Non-communicable diseases
SES	Socioeconomic status

## References

[1] World Health Organisation (WHO) Cardiovascular Diseases (CVDs). https://www.who.int/news-room/fact-sheets/detail/cardiovascular-diseases-(cvds).

[2] Wang X., Zeng Q., Ma N., Peng L., Liu L., Hong F., Xu Y. (2023). Sex-specific differences in the association between metabolically healthy overweight/obesity and the risk of hypertension in Chinese ethnic minorities. Endocrine.

[3] Gafane-Matemane L.F., Craig A., Kruger R., Alaofin O.S., Ware L.J., Jones E.S.W., Kengne A.P. (2024). Hypertension in sub-Saharan Africa: The current profile, recent advances, gaps, and priorities. J. Hum. Hypertens..

[4] Pandey K.N. (2024). Genetic and Epigenetic Mechanisms Regulating Blood Pressure and Kidney Dysfunction. Hypertension.

[5] Zhou B., Carrillo-Larco R.M., Danaei G., Riley L.M., Paciorek C.J., Stevens G.A., Gregg E.W., Bennett J.E., Solomon B., Singleton R.K. (2021). Worldwide trends in hypertension prevalence and progress in treatment and control from 1990 to 2019: A pooled analysis of 1201 population-representative studies with 104 million participants. Lancet.

[6] Olowoyo P., Okekunle A.P., Asowata O.J., Atolani S., Morsy M.I., Caiazzo E., Gaye B., Kadan D.D., Bruzzese D., Guzik T.J. (2025). Prevalence of hypertension in Africa in the last two decades: Systematic review and meta-analysis. Cardiovasc. Res..

[7] Gómez-Olivé F.X., Ali S.A., Made F., Kyobutungi C., Nonterah E., Micklesfield L., Alberts M., Boua R., Hazelhurst S., Debpuur C. (2017). Regional and Sex Differences in the Prevalence and Awareness of Hypertension: An H3Africa AWI-Gen Study Across 6 Sites in Sub-Saharan Africa. Glob. Heart.

[8] Woodiwiss A.J., Orchard A., Mels C.M.C., Uys A.S., Nkeh-Chungag B.N., Kolkenbeck-Ruh A., Ware L.J., Yates S., Jones E.S.W., Peterson V.R. (2023). High prevalence but lack of awareness of hypertension in South Africa, particularly among men and young adults. J. Hum. Hypertens..

[9] Sharma J.R., Mabhida S.E., Myers B., Apalata T., Nicol E., Benjeddou M., Muller C., Johnson R. (2021). Prevalence of hypertension and its associated risk factors in a rural black population of mthatha town, South Africa. Int. J. Environ. Res. Public Health.

[10] Kandala N.B., Nnanatu C.C., Dukhi N., Sewpaul R., Davids A., Reddy S.P. (2021). Mapping the burden of hypertension in south africa: A comparative analysis of the national 2012 sanhanes and the 2016 demographic and health survey. Int. J. Environ. Res. Public Health.

[11] Remais J.V., Zeng G., Li G., Tian L., Engelgau M.M. (2013). Convergence of non-communicable and infectious diseases in low- and middle-income countries. Int. J. Epidemiol..

[12] Berry K.M., Parker W.-A., Mchiza Z.J., Sewpaul R., Labadarios D., Rosen S., Stokes A. (2017). Quantifying unmet need for hypertension care in South Africa through a care cascade: Evidence from the SANHANES, 2011–2012. BMJ Glob. Health.

[13] Kaswa R., de Villiers M. (2020). Prevalence of substance use amongst people living with human immunodeficiency virus who attend primary healthcare services in Mthatha, South Africa. S. Afr. Fam. Pract..

[14] World Medical Association (2013). World Medical Association Declaration of Helsinki: Ethical principles for medical research involving human subjects. JAMA.

[15] World Health Organisation (WHO) (2008). WHO|Waist Circumference and Waist–Hip Ratio. Report of a WHO Expert Consultation.

[16] Seedat Y.K., Rayner B.L., Veriava Y. (2014). South African hypertension practice guideline 2014. Cardiovasc. J. Afr..

[17] Mphekgwana P.M., Malema N., Monyeki K.D., Mothiba T.M., Makgahlela M., Kgatla N., Makgato I., Sodi T. (2020). Hypertension prevalence and determinants among black South African adults in semi-urban and rural areas. Int. J. Environ. Res. Public Health.

[18] Peer N., Steyn K., Lombard C., Gwebushe N., Levitt N. (2013). A high burden of hypertension in the urban black population of Cape Town: The Cardiovascular Risk in Black South Africans (CRIBSA) study. PLoS ONE.

[19] Ntuli S.T., Maimela E., Alberts M., Choma S., Dikotope S. (2015). Prevalence and associated risk factors of hypertension amongst adults in a rural community of Limpopo Province, South Africa. Afr. J. Prim. Health Care Fam. Med..

[20] (2013). GBD 2013 Risk Factors Collaborators. Global, regional, and national comparative risk assessment of 79 behavioural, environmental and occupational, and metabolic risk factors or clusters of risks in 188 countries, 1990–2013: A systematic analysis for the Gl. Lancet.

[21] Giri A., Hellwege J.N., Keaton J.M., Park J., Qiu C.X., Warren H.R., Torstenson E.S., Kovesdy C.P., Sun Y.V., Wilson O.D. (2019). Trans-ethnic association study of blood pressure determinants in over 750,000 individuals. Nat. Genet..

[22] Afrifa-Anane E., Agyemang C., Codjoe S.N.A., Ogedegbe G., De-Graft Aikins A. (2015). The association of physical activity, body mass index and the blood pressure levels among urban poor youth in Accra, Ghana. BMC Public Health.

[23] Forouzanfar M.H., Afshin A., Alexander L.T., Anderson H.R., Bhutta Z.A., Biryukov S., Brauer M., Burnett R., Cercy K., Charlson F.J. (2016). Global, regional, and national comparative risk assessment of 79 behavioural, environmental and occupational, and metabolic risks or clusters of risks, 1990–2015: A systematic analysis for the Global Burden of Disease Study 2015. Lancet.

[24] Dhanachandra Singh K., Jajodia A., Kaur H., Kukreti R., Karthikeyan M. (2014). Gender specific association of RAS gene polymorphism with essential hypertension: A case-control study. BioMed Res. Int..

[25] Wandai M.E., Norris S.A., Aagaard-Hansen J., Manda S.O.M. (2020). Geographical influence on the distribution of the prevalence of hypertension in South Africa: A multilevel analysis. Cardiovasc. J. Afr..

[26] Carretero O.A., Oparil S. (2000). Essential Hypertension Part I: Definition and Etiology Clinical Cardiology: New Frontiers. Circulation.

[27] Lionakis N., Mendrinos D., Sanidas E., Favatas G., Georgopoulou M. (2012). Hypertension in the elderly. World J. Cardiol..

[28] Wang H., Naghavi M., Allen C., Barber R.M., Bhutta Z.A., Carter A., Casey D.C., Charlson F.J., Chen A.Z., Coates M.M. (2016). Global, regional, and national life expectancy, all-cause mortality, and cause-specific mortality for 249 causes of death, 1980–2015: A systematic analysis for the Global Burden of Disease Study 2015. Lancet.

[29] Alberts M., Urdal P., Steyn K., Stensvold I., Tverdal A., Nel J.H., Steyn N.P. (2005). Prevalence of cardiovascular diseases and associated risk factors in a rural black population of South Africa. Eur. J. Prev. Cardiol..

[30] Cornelissen V.A., Smart N.A. (2013). Exercise training for blood pressure: A systematic review and meta-analysis. J. Am. Heart Assoc..

[31] Neter J.E., Stam B.E., Kok F.J., Grobbee D.E., Geleijnse J.M. (2003). Influence of Weight Reduction on Blood Pressure: A Meta-Analysis of Randomized Controlled Trials. Hypertension.

[32] Roerecke M., Kaczorowski J., Tobe S.W., Gmel G., Hasan O.S.M., Rehm J. (2017). The effect of a reduction in alcohol consumption on blood pressure: A systematic review and meta-analysis. Lancet Public Health.

[33] The SPRINTResearch Group (2015). ARandomized Trial of Intensive versus Standard Blood-Pressure Control. N. Engl. J. Med..

[34] Spence J.D. (2018). Controlling Resistant Hypertension.

[35] Goit L.N., Yang S. (2019). Treatment of Hypertension: A Review. Yangtze Med..

[36] Cheung B.M.Y., Li C. (2012). Diabetes and hypertension: Is there a common metabolic pathway?. Curr. Atheroscler. Rep..

[37] Adeniyi O.V., Yogeswaran P., Longo-Mbenza B., Ter Goon D. (2016). Uncontrolled hypertension and its determinants in patients with concomitant type 2 diabetes mellitus (T2DM) in rural South Africa. PLoS ONE.

[38] Shukla A., Kumar K., Singh A. (2014). Association between obesity and selected morbidities: A study of BRICS countries. PLoS ONE.

[39] Alaba O., Chola L. (2014). Socioeconomic inequalities in adult obesity prevalence in South Africa: A decomposition analysis. Int. J. Environ. Res. Public Health.

[40] Lozano R., Naghavi M., Foreman K., Lim S., Shibuya K., Aboyans V., Abraham J., Adair T., Aggarwal R., Ahn S.Y. (2012). Global and regional mortality from 235 causes of death for 20 age groups in 1990 and 2010: A systematic analysis for the Global Burden of Disease Study 2010. Lancet.

[41] Adeniyi O.V., Longo-Mbenza B., Ter Goon D. (2015). Female sex, poverty and globalization as determinants of obesity among rural South African type 2 diabetics: A cross-sectional study. BMC Public Health.

[42] Benkeser R.M., Biritwum R., Hill A.G. (2012). Prevalence of overweight and obesity and perception of healthy and desirable body size in urban, Ghanaian women. Ghana Med. J..

[43] Bell A.C., Adair L.S., Popkin B.M. (2004). Understanding the role of mediating risk factors and proxy effects in the association between socio-economic status and untreated hypertension. Soc. Sci. Med..

[44] Leng B., Jin Y., Li G., Chen L., Jin N. (2015). Socioeconomic status and hypertension: A meta-analysis. J. Hypertens..

[45] Kagura J., Adair L.S., Pisa P.T., Griffiths P.L., Pettifor J.M., Norris S.A. (2016). Association of socioeconomic status change between infancy and adolescence, and blood pressure, in South African young adults: Birth to Twenty cohort. BMJ Open.

[46] Poti J.M., Braga B., Qin B. (2017). Ultra-processed Food Intake and Obesity: What Really Matters for Health-Processing or Nutrient Content?. Curr. Obes. Rep..

[47] Mayén A.L., Bovet P., Marti-Soler H., Viswanathan B., Gedeon J., Paccaud F., Marques-Vidal P., Stringhini S. (2016). Socioeconomic differences in dietary patterns in an East African country: Evidence from the Republic of Seychelles. PLoS ONE.

[48] Zajacova A., Lawrence E.M. (2018). The Relationship between Education and Health: Reducing Disparities Through a Contextual Approach. Annu. Rev. Public Health.

[49] Seow L.S.E., Subramaniam M., Abdin E., Vaingankar J.A., Chong S.A. (2015). Hypertension and its associated risks among Singapore elderly residential population. J. Clin. Gerontol. Geriatr..

